# Machine Learning-Based Human Detection Using Active Non-Line-of-Sight Laser Sensing

**DOI:** 10.3390/s26072046

**Published:** 2026-03-25

**Authors:** Semra Çelebi, İbrahim Türkoğlu

**Affiliations:** 1 Faculty of Engineering, Department of Computer Engineering, Siirt University, Siirt 56000, Türkiye; 2 Faculty of Technology, Department of Software Engineering, Fırat University, Elazığ 23000, Türkiye; iturkoglu@firat.edu.tr

**Keywords:** NLOS, non-line of sight, laser signals, deep learning, remote sensing

## Abstract

Active non-line-of-sight (NLOS) human detection aims to infer the presence of hidden individuals by analyzing indirectly reflected photons between a relay surface and occluded targets. In this study, a single-photon avalanche diode (SPAD) and time-correlated single-photon counting (TCSPC)-based acquisition system were used to measure time–photon waveforms in controlled NLOS environments designed to represent post-disaster rubble scenarios. Although the effective temporal resolution of the system is limited by the detector timing jitter and laser pulse width, the recorded transient signals retain distinguishable intensity and temporal delay patterns associated with the primary and secondary reflections. To construct a representative dataset, measurements were collected under varying subject poses, orientations, and surrounding object configurations. The recorded signals were processed using a unified preprocessing pipeline that included normalization, histogram shaping, and signal windowing. Three machine learning models, namely, Convolutional Neural Network, Gated Recurrent Unit, and Random Forest, were trained and evaluated for human presence classification. All models achieved full sensitivity in detecting human presence; however, notable differences emerged in the classification of human-absent scenarios. Among the tested approaches, random forest achieved the highest overall accuracy and specificity, demonstrating stronger robustness to statistical variations in time–photon histograms under limited photon conditions. These results suggest that tree-based classifiers capture amplitude distribution patterns and temporal dispersion characteristics more effectively than deep neural architectures under the present acquisition constraints. Overall, the findings indicate that low-cost SPAD-based NLOS sensing systems can provide reliable human detection in indirect-observation scenarios.

## 1. Introduction

Non-line-of-sight (NLOS) sensing has emerged as a critical research frontier for extracting information from hidden scenes behind obstacles. While conventional imaging systems excel in line-of-sight (LOS) conditions, their performance significantly degrades when the direct light path is obstructed. This limitation necessitates robust NLOS approaches for high-stakes applications such as search and rescue, security, and surveillance.

The primary challenge in NLOS systems is extracting meaningful information from signals whose structural characteristics are altered by multiple interactions with the environment, including reflection, refraction, and absorption. Recently, machine learning-based methods have proven effective in addressing these complexities, offering faster and more stable analysis compared to traditional reconstruction techniques.

NLOS sensing is generally categorized into passive and active methods. Passive techniques utilize ambient signals (e.g., thermal emissions, natural light), requiring minimal hardware but suffering from low spatial resolution and environmental sensitivity. Conversely, active methods illuminate the scene with controlled signals, providing higher resolution and superior signal-to-noise ratios. In active sensing, capturing transient light transport is vital. Single-photon avalanche diodes (SPADs), combined with time-correlated single-photon counting (TCSPC), are widely adopted for their picosecond-level temporal resolution, enabling the extraction of distance and geometric information from time–photon histograms.

Despite recent progress, several limitations remain in the literature. First, many studies rely heavily on simulation environments [1,2,3,4], which often fail to account for hardware noise and real-world uncertainties. Second, experimental studies typically focus on static objects [5,6,7], while human detection—particularly involving dynamic subjects—remains underexplored [2,8]. Finally, although neural networks (e.g., Convolutional Neural Networks (CNNs) and Recurrent Neural Networks (RNNs), including Gated Recurrent Units (GRU)) and classical machine learning approaches such as Random Forest (RF) provide powerful feature extraction capabilities, comparative evaluations using real SPAD-TCSPC measurements remain limited. Neural network models often require experimentally collected data to function effectively [9]. Consequently, the scarcity of scenario-based NLOS datasets involving human subjects remains a significant limitation.

This study addresses these gaps by evaluating learning-based approaches using real experimental measurements. We designed an active SPAD-TCSPC system and collected data from multiple subjects in diverse settings. The main contributions of this study are summarized as follows:

- Development of a new experimental active NLOS detection system based on SPAD and TCSPC.
- Creation of a scenario-focused NLOS dataset collected from different human participants in separate measurements.
- A comparative evaluation of CNN, GRU, and RF models using real-world measurements.
- An analysis of model generalization and robustness under varying environmental conditions.

The remainder of this paper is organized as follows. Section 2 reviews related studies on NLOS sensing, including active NLOS systems and learning-based approaches. Section 3 describes the materials and methods used in this study, including the proposed active NLOS system and data acquisition process, data preprocessing and dataset construction, the learning models, and the experimental setup and evaluation protocol. Section 4 presents the experimental results, including the performance of the CNN, GRU, and RF models and their overall comparative analysis. Section 5 discusses the obtained results, and Section 6 concludes the study and outlines directions for future work.

## 2. Related Work

NLOS detection focuses on analyzing signals that return to a sensor through indirect paths after interacting with environmental obstacles. In the literature, acoustic signals have been extensively explored as an alternative to optical sensing. For instance, acoustic reflections have been utilized to estimate the position of hidden sources in autonomous vehicles and robotic platforms [10,11]. This modality also enables material-based object classification using multi-channel systems [12], vehicle detection through microphone arrays [13], and even 3D reconstruction of structures behind walls by combining multi-viewpoint measurements [7]. However, acoustic propagation remains highly sensitive to environmental conditions and suffers from limited spatial resolution in cluttered scenes.

To overcome these limitations, optical NLOS methods—categorized as passive and active—have gained prominence. Passive techniques exploit ambient light, shadows, or spectral cues to infer hidden-scene information. Research has demonstrated that wall edges can function as “passive cameras” to encode motion [14], while subsequent studies established that 2D/3D scene recovery remains feasible even with unknown occluder geometries [15,16]. Other strategies include the analysis of shadows and indirect illumination for indoor scene inference [17,18], as well as the use of multimodal constraints and stereo configurations to enhance reconstruction robustness [19,20,21]. Notably, the HiddenPose framework [22] recently demonstrated the recovery of articulated human-like structures for pose estimation.

Despite these advancements, passive methods are fundamentally constrained by their reliance on favorable, uncontrolled illumination and specific scene assumptions, which often fail in highly attenuated or complex environments. Consequently, active NLOS approaches have become the preferred solution for robust sensing. By utilizing controlled illumination and time-resolved hardware, active systems generate stable photon-count histograms that are inherently more resilient to noise and environmental variability. Following this trajectory, the present study adopts an active NLOS framework, focusing on the learning-based classification of human presence using real-world experimental data.

### 2.1. Active NLOS Systems

**Figure 1:**
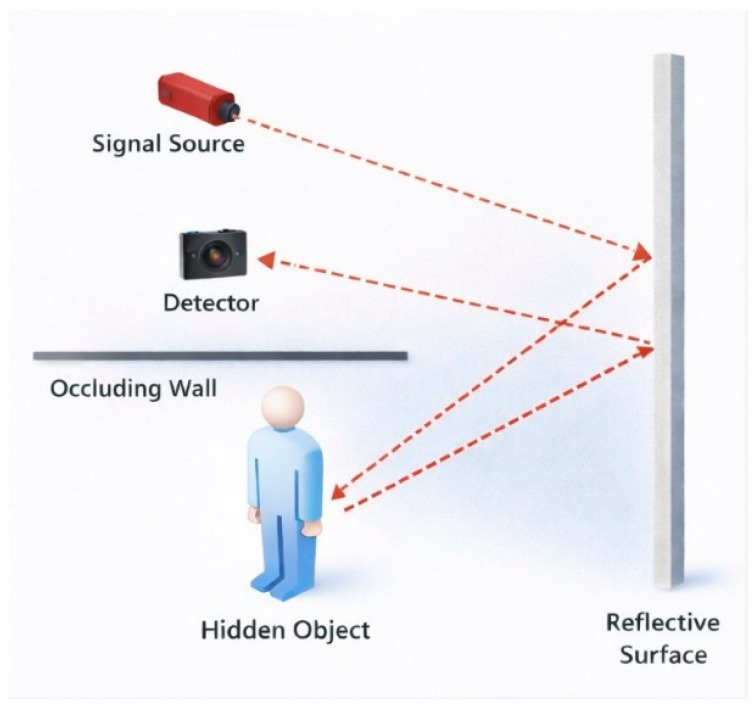
Active non-line-of-sight system.

Active NLOS systems use controlled illumination to infer information about scenes hidden behind obstacles. These systems rely on multi-bounce light propagation, where emitted light first reaches a relay surface, interacts with the hidden scene, and returns to the detector after successive reflections (Figure 1). This indirect propagation enables recovery of information about objects outside the line of sight from time-resolved measurements [23]. The returned signals are analyzed using computational approaches such as back-projection [24,25], inverse reconstruction methods [26], and wave-based propagation models [27,28].

Laser-based optical systems are commonly used in active NLOS sensing because they provide controlled illumination and precise temporal measurements. Time-of-flight (ToF) analysis measures photon arrival times from primary and secondary reflections. In many implementations, SPAD detectors combined with TCSPC electronics capture these signals as time–photon histograms representing the temporal structure of the returned light.

Various acquisition strategies have been proposed for SPAD-based NLOS sensing. Galvanometer scanning systems provide flexible spatial sampling but are typically limited to speeds of 1–10 Hz [23], increasing acquisition time for high-resolution grids [28,29]. To reduce this limitation, studies have explored reducing scan points [30,31], optimizing scan trajectories [32], or using scanner-free SPAD array architectures [27,33,34]. In this study, a 50 × 50 scanning matrix provides a practical compromise between spatial sampling density and acquisition time.

Advances in SPAD detector technology have also improved spatial resolution and timing accuracy. Early work investigated timing accuracy using gated 16 × 1 SPAD arrays [35], while later studies introduced microlens-enhanced 16 × 16 arrays to improve photon collection efficiency [5]. Comparative analyses highlighted the influence of detector resolution and timing precision on detection performance [1]. Larger arrays have enabled motion detection and depth mapping using 128 × 128 SPAD sensors [36], while large-scale systems using InGaAs/InP SPAD detectors and confocal telescope designs demonstrated kilometer-scale NLOS tracking [37].

ToF-based NLOS systems have also shown strong capabilities for scene reconstruction and depth estimation. For instance, SPAD-based ToF systems operating at near-infrared wavelengths have enabled depth mapping, object tracking, and material classification [38]. Interferometric ToF approaches implemented with CCD/CMOS sensors achieved dynamic three-dimensional measurements with sub-millimeter accuracy using synthetic wavelengths [39]. However, scanner-free and array-based configurations may still face limitations related to detector fill factor, calibration requirements, and timing uncertainties [33].

A preliminary study closely related to the sensing architecture used here was presented by Olgun et al. [40], who detected human presence in NLOS environments using primary and secondary reflections from a low-cost laser-SPAD system. Despite limited temporal resolution, the study achieved approximately 76% classification accuracy, demonstrating that photon density and temporal delay patterns contain useful information for distinguishing human presence.

Despite these advances, many SPAD-TCSPC-based studies remain limited to static scenes and controlled experimental conditions [41,42,43], with most research focusing on reconstruction rather than detection tasks [44,45,46]. Experimental studies involving living subjects and diverse scenarios are still relatively limited [14,47,48]. Therefore, this study adopts an active SPAD-TCSPC sensing framework and investigates human detection using a scenario-based dataset collected under diverse experimental conditions. By combining real measurements with learning-based analysis, the proposed approach aims to extend experimental NLOS datasets while maintaining a widely used sensing architecture.

### 2.2. Learning-Based Approaches for NLOS

Although active NLOS systems provide robust sensing infrastructures, interpreting the resulting high-dimensional and time-resolved photon data remains challenging. Time–photon histograms generated by indirect reflections often exhibit complex temporal structures that are difficult to analyze using conventional signal-processing methods. Consequently, learning-based approaches have emerged as effective tools for extracting discriminative patterns from these measurements. Artificial intelligence models can learn both spatial and temporal characteristics of reflected signals directly from data, enabling improved interpretation of NLOS measurements.

CNNs have demonstrated strong performance in extracting complex patterns from high-dimensional data [49,50]. RNNs, particularly GRU architectures, are well suited for modeling sequential dependencies in time-series signals and have shown strong performance in temporal signal analysis [51]. In addition to deep learning approaches, classical machine learning algorithms such as decision tree-based models remain valuable due to their interpretability, low computational cost, and stable performance in structured classification problems. These characteristics motivate the evaluation of multiple learning paradigms for NLOS signal analysis.

Several studies have applied deep learning to NLOS imaging and reconstruction. Lei et al. [2] demonstrated that CNN architectures such as SimpleNet and ResNet-18 can recognize hidden objects from laser-based speckle patterns. Metzler et al. [52] further showed that a U-Net architecture can outperform classical reconstruction methods under low signal-to-noise conditions.

Sequential learning models have also been explored for time-resolved photon data. Lin et al. [53] analyzed SPAD-TCSPC measurements using a GRU-based network that directly processes photon timestamps without explicit histogram construction. Similarly, Isogawa et al. [8] proposed a deep learning framework for three-dimensional human pose estimation from transient NLOS measurements, combining feature extraction from transient signals with recurrent architectures to model temporal dependencies.

More recently, transformer-based models have been introduced for NLOS reconstruction tasks. Architectures such as NLOST [54] and MARMOT [55] utilize attention mechanisms to capture global spatial relationships and reconstruct scenes from sparse or irregular measurements. However, most learning-based NLOS studies focus primarily on reconstruction using a single model architecture, and systematic comparisons across different learning paradigms remain limited. Earlier reviews, such as Faccio et al. [56], highlighted the growing role of machine learning in NLOS imaging, yet subsequent work has largely emphasized convolutional reconstruction pipelines.

Despite these advances, several gaps remain. Many studies focus on scene reconstruction rather than detection or classification tasks, while the sequential temporal structure of time–photon histograms remains relatively underexplored. Moreover, classical ensemble-based methods are rarely compared systematically with deep neural architectures. These limitations motivate the present study, which investigates multiple learning approaches for analyzing time–photon signals obtained from real SPAD-TCSPC measurements.

Therefore, this work performs a comparative evaluation of CNN, GRU-based RNN, and RF models using the same data representation, preprocessing pipeline, and evaluation protocol. By analyzing real experimental measurements rather than simulated datasets, the proposed approach provides a more comprehensive assessment of learning-based methods for human-presence detection in active NLOS scenarios.

## 3. Materials and Methods

This section presents the active NLOS measurement system, the acquisition protocol used to collect SPAD-TCSPC transient data, the preprocessing steps applied to the signals, and the machine learning models employed in the experimental evaluation.

### 3.1. Proposed Active NLOS System and Data Acquisition

**Figure 2:**
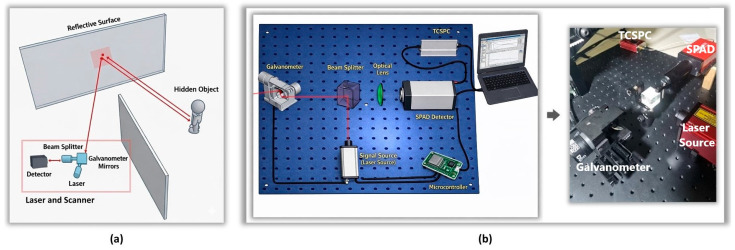
Experimental NLOS laboratory setup: (**a**) Geometry of the controlled laboratory environment; (**b**) Optical acquisition system including the pulsed laser, beam splitter, galvanometer scanner, SPAD detector, and TCSPC module.

Within the scope of this study, a controlled and isolated laboratory environment has been designed to enable the execution of active NLOS laser experiments. The geometry of the experimental setup was configured to indirectly illuminate the hidden scene via a visible reflective surface and to detect photons returning through secondary reflections. The general architecture of the established system and the light propagation path are shown schematically in Figure 2a.

During the setup of the environment, the goal was to minimize the effects of ambient light, unwanted optical reflections, and noise signals that could negatively affect the accuracy of the measurements. In this context, the experimental area was isolated from external influences using appropriate protective and insulating materials. The experiments were conducted in an insulated laboratory area measuring 2.6 m × 3 m. The ceiling of the working area was covered with furniture materials to reduce optical reflections. A barrier measuring 2 m × 2 m was placed inside the experimental area. This structure was positioned to create hidden stage conditions and block the direct line of sight. The surface of the barrier was covered with insulation materials to prevent light leakage during the experiments. Additionally, to prevent unwanted reflections of laser beams from surfaces and to absorb ambient lighting, the perimeter of the experimental area was completely enclosed with seamless blackout curtains.

To detect a living presence in a NLOS environment, primary and secondary reflected laser signals were used in accordance with existing studies in the literature. An optical measurement system, as shown in Figure 2b, was established for this purpose. The system comprises a pulsed laser source (Thorlabs, Newton, NJ, USA; model: NPL64B), polarized beam splitter (Thorlabs, Newton, NJ, USA; model: PBS251), galvanometer-based scanning mechanism (Thorlabs, Newton, NJ, USA; model: GVS012/M), SPAD detector (Thorlabs, Newton, NJ, USA; model: SPDMH2), and TCSPC module (Thorlabs, Newton, NJ, USA; model: SPCNT).

The pulsed laser operated at a wavelength of 640 nm with an output power of 35 mW and a repetition rate of 10 MHz, corresponding to a pulse period of 100 ns. Photon detection was performed using a SPAD, providing a timing jitter of approximately 350 ps (FWHM) and a typical dead time of approximately 60 ns. Time-resolved photon counting was achieved using a TCSPC module operating in internal binning mode. The effective temporal bin resolution was approximately 5 ps per bin, resulting in 20,000 time bins spanning a total acquisition window of approximately 100 ns per scanning point.

Although 20,000 temporal bins were recorded per excitation cycle, only the physically relevant portion of the histogram containing significant photon returns was retained during preprocessing, resulting in effective signal lengths of approximately 16,218 samples prior to alignment and standardization. The effective system temporal resolution, determined by the combined effects of laser pulse width and detector timing jitter, was on the order of several hundred picoseconds.

During the experiments, the laser beam was directed toward the relay reflective surface using a galvanometer-based scanning mechanism. Photons returning indirectly from the hidden scene were detected by the SPAD sensor, and the corresponding photon arrival times were recorded through the TCSPC module. Figure 2 illustrates the experimental NLOS laboratory setup.

To scan the experimental environment, the laser beam was projected across a 50 × 50 scanning grid on the reflective surface using the galvanometer system. At each scanning position, photon signals were collected for 1 ms to accumulate photon statistics. After each acquisition period, an additional 4 ms waiting interval was introduced to ensure detector recovery and measurement stability before moving to the next scanning point. This procedure improved measurement repeatability and enhanced the consistency of the acquired data.

**Figure 3:**
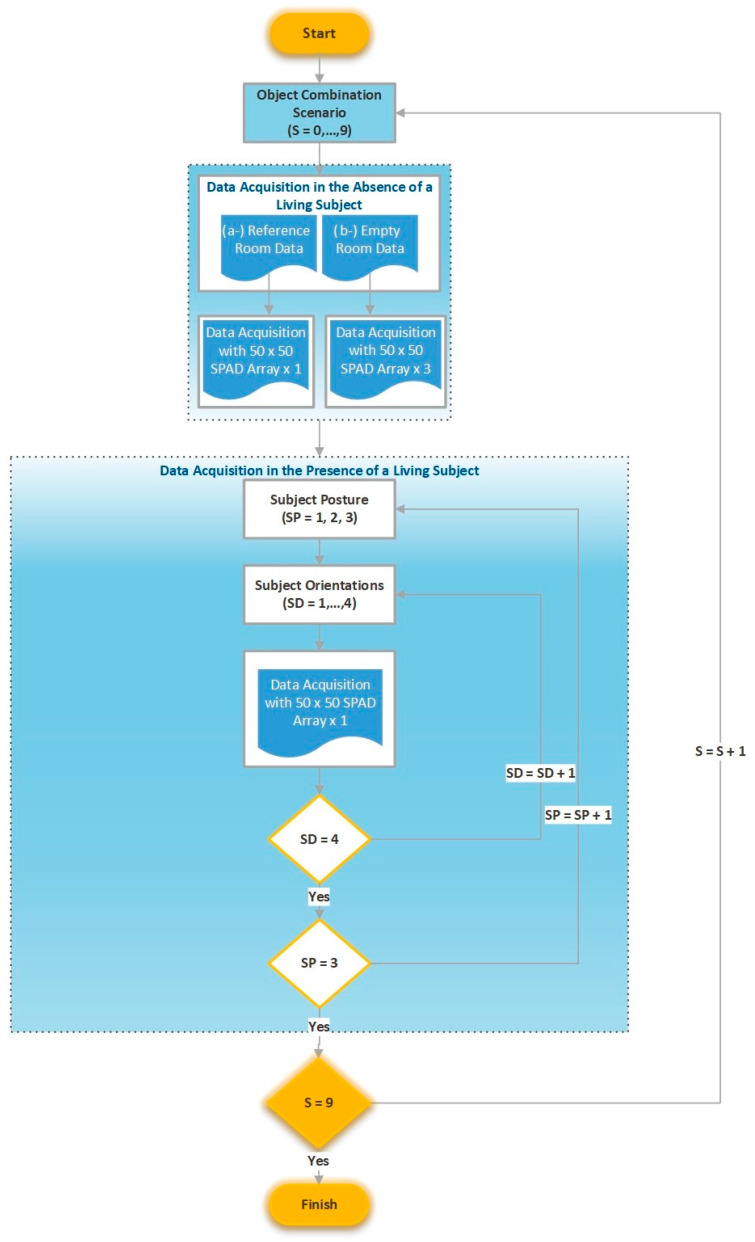
Laser signal data acquisition workflow diagram.

After the alignment of the optical components and stabilization of the system, the experimental data acquisition procedure shown in Figure 3 was implemented.

Step 1: An object scenario located in a NLOS environment was created by considering materials that are highly likely to be present near a living being during a debris situation. In this context, five types of objects commonly found in debris environments wood, iron, brick, PVC, and household appliances were selected. Ten different scenarios, formed from single and paired combinations of these objects, were configured, as shown in Table 1. These scenarios were used to test the consistency of the signal characteristics related to human presence in different object environments.

Step 2: In the absence of a human subject, NLOS measurements were first recorded for each object configuration to obtain background reflection signals. For each scenario, one reference room dataset (no objects present) and three empty room datasets (object present but no human subject) were recorded to ensure measurement stability and dataset diversity.

- Reference Room Data: Reflected laser signals obtained when neither objects nor humans were present in the hidden scene. These measurements were used as baseline calibration data to compensate for gradual signal amplitude variations caused by factors such as laser source heating.
- Empty Room Data: Reflected signals recorded when an object scenario was present but no human subject was located in the hidden scene. Each object configuration was measured three times to increase the diversity of the human-absent dataset used during model training.

**Figure 4:**
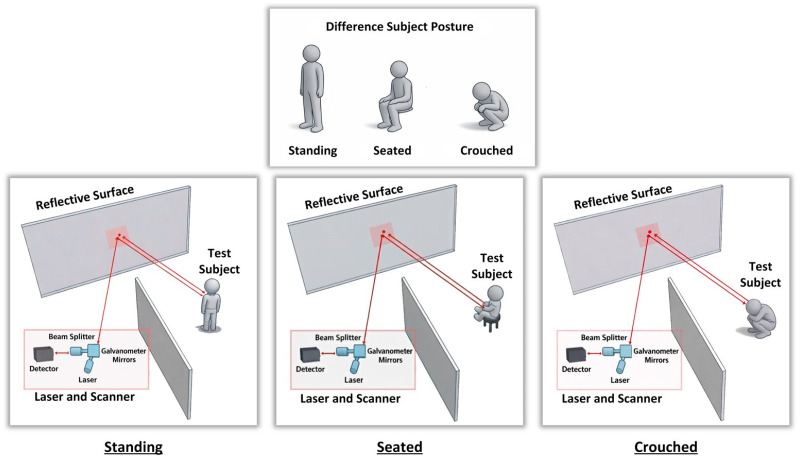
Three different posture positions of the test subject: standing, seated, and crouched.

**Figure 5:**
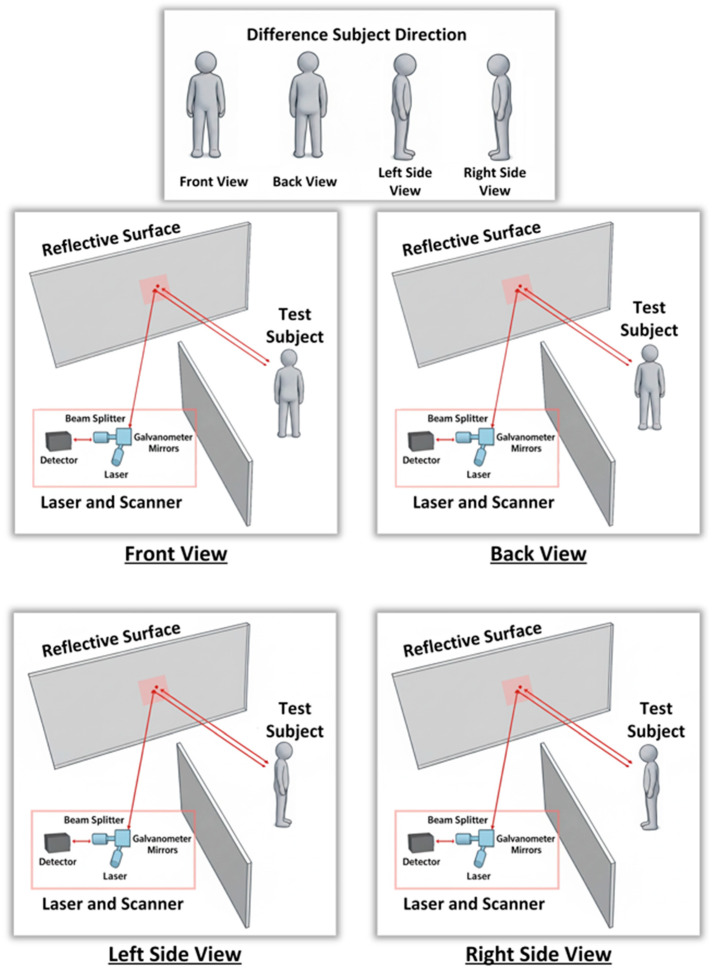
Four different whole-body orientations of the test subject relative to the reflective surface: front side, back side, right side, and left side.

Step 3: When a living being was present in the out-of-line-of-sight environment, laser signals were transmitted to the scene and data was collected. The data collection process was carried out as shown in Figure 4, considering three different posture positions of the living subjects (standing, sitting, and crouching) and four different orientations relative to the reflective surface (front, back, right, and left) for each posture, as shown in Figure 5. In this context, a total of 12 data sets were obtained for each living subject, each consisting of a reflected laser signal comprising 2500 points. During the experimental setup, the spatial positions of both the human subjects and the object configurations were predefined and kept constant across all scenarios to ensure measurement repeatability and controlled comparison. In each scenario, subjects were placed at the same predefined coordinates relative to the relay reflective surface. To minimize variability arising from clothing-related reflections, participants wore white sleeveless garments that exposed most of the upper body surface. Reflective accessories such as jewelry, piercings, glasses, belts, or other metallic objects were not permitted during the measurements. Female participants were asked to tie their hair to increase exposed body surface area and reduce uncontrolled scattering effects. These controlled conditions were adopted to isolate the reflection characteristics of the human body and to reduce potential confounding effects introduced by external materials. The relay surface consisted of a flat aluminum panel with high reflectivity, providing stable and consistent specular-dominant reflections throughout the experiments.

Step 4: The data collection process was repeated on 10 living subjects. The subject group was selected to consist of 5 female and 5 male individuals.

### 3.2. Data Preprocessing and Dataset Construction

**Figure 6:**
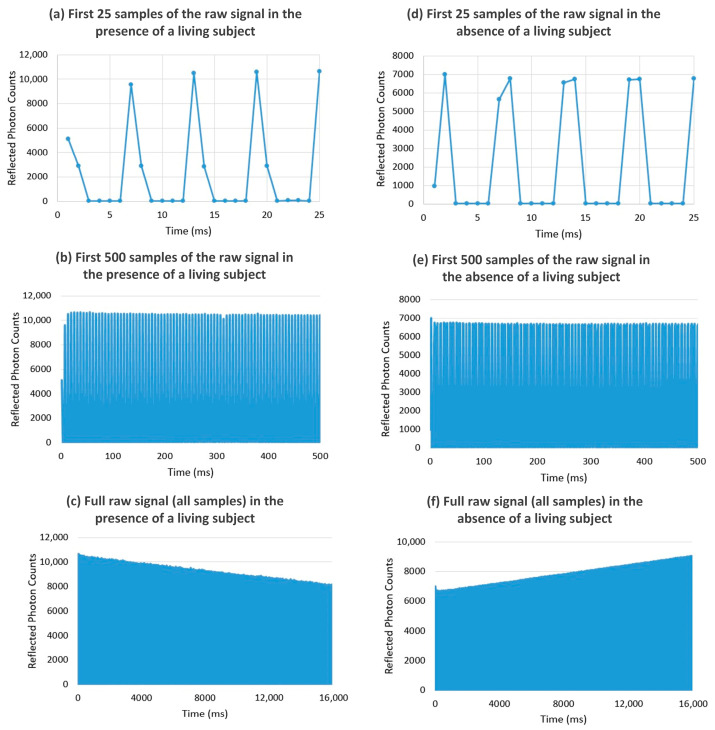
Raw laser reflection signals obtained under the same object scenario for both the presence and absence of a living subject.

Within the scope of this study, 1200 data samples labeled as human-present and 400 data samples labeled as human-absent were collected. For each experiment, time-resolved photon-counting data were obtained for 50 × 50 scanning points. Because the SPAD-TCSPC system records the photon arrival times at each scanning point over thousands of time bins, the raw data have a time–photon histogram structure. Therefore, the recorded data contained approximately six time bins, and the total number of data rows for a single experiment was 16,218. Figure 6 shows the raw laser reflection signals obtained in the presence and absence of a living being under the same object scenario. The gradual envelope variations observed in some full-length signals are attributed to acquisition-related drift effects and occur in both classes; therefore, they are not indicative of human presence. The discriminative information lies in the reflection photon count distribution, particularly in the relative amplitudes and temporal spread of primary and secondary reflections. In human-present scenarios, additional scattering and absorption subtly modify photon redistribution patterns. Although these differences are not reliably distinguishable through visual inspection, they are captured effectively by the proposed learning models.

It was observed that in the dataset, some raw signals started from the peak regions of the signal while others began from the background region. To ensure data consistency, the first significant peak in each signal was detected and used for temporal alignment. An empirical threshold of 1000 photons was selected based on the observed photon-count distribution in the dataset, where background counts remained relatively low while reflection peaks produced a substantial increase in photon counts. This threshold enabled reliable identification of the onset of the first strong reflection peak across the signals. The time axis of each signal was then shifted such that the time sample corresponding to this detected peak became the starting point of the signal. After time-axis alignment, some signals exhibited missing time bins toward the end. To ensure equal length across all signals, the missing time bins were filled with zeros, thereby standardizing the data size. Zero-padding was applied solely to eliminate the length discrepancies between the signals. The added padding values constituted only a very small portion of the total signal length (at most 1–2 samples). This operation, which is negligible compared to the overall data size, does not affect the distribution characteristics of the signals. Following time alignment and length standardization, all signals were normalized using the z-score method to eliminate amplitude differences. Each signal was converted to a zero-mean, unit variance format by subtracting the mean and dividing it by the standard deviation. Z-score normalization was preferred to eliminate the amplitude and scale differences between the signals obtained under varying experimental conditions. Thus, the signals were made comparable solely in terms of their structural characteristics, enabling the artificial intelligence model to be trained in a more balanced and stable manner.

While creating the dataset, three empty environment data samples were recorded for each object scenario in which a living being was not present in the NLOS environment. In contrast, considering the three different postural positions and four different orientations of the living subject, a total of 12 human-present labeled data samples were obtained for each living-object scenario. Therefore, for 10 object scenarios and 10 living subjects, 400 human-absent labeled data samples and 1200 human-present labeled data samples were recorded; the resulting class imbalance required additional balancing steps during the training process. To address this imbalance, the human-absent labeled dataset was augmented using signal-preserving transformations that introduce controlled measurement variability while maintaining the intrinsic physical structure of the recorded signals. In this process, four different data augmentation techniques were applied that did not distort the peak points or the fundamental characteristics of the signals.

- Time Shifting: Small temporal shifts were introduced by shifting the signal to the left or right by a certain number of samples to emulate minor timing misalignments in the acquisition process.
- Gain Jitter: The signal amplitude was multiplied by a randomly selected small coefficient to mimic the amplitude variations that may arise from the measurement conditions (e.g., minor intensity fluctuations in photon-count measurements).
- Low-Frequency Trend Addition (Jitter Trend): A low-frequency small trend or parabolic drift was added to the signal to model slow measurement drift or system-related variations (e.g., gradual baseline changes) rather than changes in physical scene conditions.
- Gaussian Noise Injection: Zero mean random Gaussian noise was added to the signal to simulate detector noise or measurement uncertainties.

**Figure 7:**
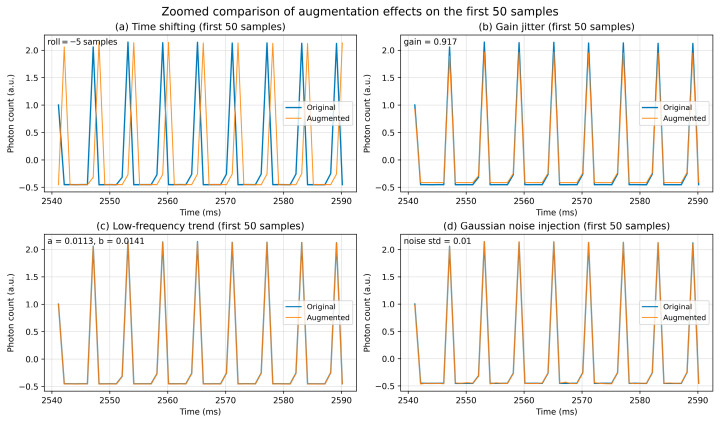
Effect of the applied data augmentation techniques on a representative time–photon signal (first 50 samples).

These operations increase the diversity of the dataset by modifying only the amplitude scale, temporal alignment, and low-level noise components of the signals, while preserving the dominant peak positions and overall signal morphology. The augmentation procedures were applied after the preprocessing stage, specifically on the Z-score normalized signals used as inputs for model training. Figure 7 illustrates a zoomed comparison of a 50-sample segment of a representative time–photon signal before and after augmentation, demonstrating that the applied transformations introduce controlled variations without altering the physical structure of the signal. Thus, the human-absent dataset was augmented, preserving the physical integrity of the signals while ensuring class balance. To ensure reliable evaluation, the dataset was partitioned using stratified sampling to reserve an independent hold-out test subset (15%) for final performance evaluation, while the remaining data were used for model training and cross-validation.

### 3.3. Learning Models

Differences in the textural properties of the subjects and surrounding objects introduce subtle variations in the internal structure of the reflected time–photon histograms. These differences are mainly reflected in the relative distribution and interaction of primary and secondary reflection components. However, they are not sufficiently distinct to allow reliable discrimination through visual inspection (Figure 6).

In addition, the long temporal span and histogram-based nature of the signals limit the effectiveness of simple threshold-based or manually engineered feature approaches. Since global envelope trends are acquisition-related and present in both classes, they do not constitute discriminative features. Therefore, data-driven learning models, including both deep learning architectures and ensemble-based classifiers, were employed to extract subtle and informative patterns directly from the measured signals.

In this study, compact 1D-CNN and GRU-based RNN architectures were designed for the classification of high-dimensional time–photon histogram signals. The models follow commonly adopted convolutional and recurrent modeling principles used in transient signal analysis [6,8,53], while the specific layer configurations, pooling strategies, and regularization settings were defined by the authors to accommodate the noise characteristics and sparsity of the experimental data. In particular, the 1D-CNN was used to capture localized temporal structures and peak characteristics within the histograms, whereas the GRU-based RNN was employed to model long-range sequential dependencies and the temporal evolution of photon arrival patterns. Prior to model training, all raw signals were standardized to a fixed length of 16,218 samples and subsequently downsampled by a factor of 2, resulting in an effective input length of 8109 time steps for the deep learning architectures. This standardization ensures dimensional consistency across samples while preserving the dominant temporal characteristics of the signal.

**Figure 8:**
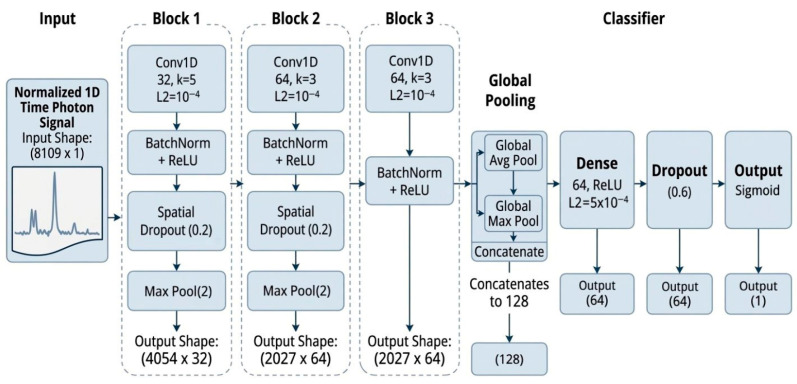
Architecture of the proposed 1D-CNN model for time–photon histogram classification.

The proposed 1D-CNN architecture (Figure 8) was designed to capture localized temporal structures such as peak characteristics, abrupt intensity transitions, and discriminative micro-patterns within time–photon histograms. The network processes normalized one-dimensional time–photon histogram signals through three convolutional blocks. The first block employs 32 filters with a kernel size of 5, followed by batch normalization, spatial dropout (0.2), and max-pooling (size 2) to reduce temporal dimensionality. The second block uses 64 filters with a kernel size of 3 together with the same normalization and regularization structure. The third block contains a convolution layer with 64 filters that extracts higher-level temporal representations without additional pooling. A hybrid global pooling layer combining global average pooling and global max pooling aggregates the resulting feature maps into a 128-dimensional representation. Finally, the classifier head consists of a fully connected layer with 64 units (ReLU activation and L2 regularization 5 × 10^−4^), followed by dropout (0.6) and a sigmoid output neuron for binary classification.

**Figure 9:**
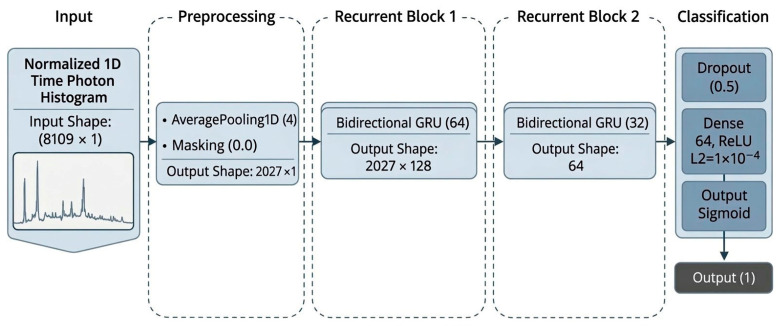
Architecture of the proposed GRU-based RNN model for time–photon histogram classification.

The GRU-based RNN architecture, illustrated in Figure 9, was implemented to model the sequential dependencies and temporal evolution of time–photon histogram signals. An initial AveragePooling1D operation reduces the temporal resolution of the input sequence, followed by a masking layer to suppress the influence of zero-padding introduced during preprocessing. The network then employs two bidirectional GRU layers, allowing the model to process the transient signal sequence in both forward and backward temporal directions. This bidirectional processing improves the representation of complex temporal dependencies by simultaneously considering earlier and later photon arrival patterns, which is particularly beneficial for capturing multipath reflections and delayed secondary peaks in NLOS photon histograms. The resulting sequential representation is subsequently processed through a regularized dense layer with dropout, followed by a sigmoid output neuron for final binary classification.

In addition to deep learning architectures, a RF classifier was incorporated as a classical ensemble-based baseline to provide a non-parametric comparison framework. The RF model was trained directly on the normalized time–photon signal vectors without additional handcrafted feature extraction, thereby preserving methodological consistency across models. To control model complexity and mitigate overfitting, several regularization-oriented hyperparameters were employed, including constrained tree depth, minimum leaf size, minimum split size, feature subsampling, and bootstrap-based bagging. The complete set of hyperparameters and their functional rationale are summarized in Table 2. This configuration enables the RF model to capture nonlinear decision boundaries while maintaining robustness against statistical variability and noise inherent in time–photon histogram signals.

### 3.4. Experimental Setup and Evaluation Protocol

To ensure methodological consistency and fair comparison across architectures, all models were trained using the same data representation and preprocessing pipeline. A total of 2400 samples (1200 human-present and 1200 human-absent) obtained from time–photon signals processed with t_0_ alignment and Z-score normalization were used for the binary classification task.

The dataset was divided into a stratified hold-out test set comprising 15% of the samples. Five-fold stratified cross-validation was applied to the remaining training data to assess model stability and guide hyperparameter selection, whereas the independent hold-out test set was reserved exclusively for final unbiased performance reporting.

For the deep learning models, training and optimization were performed according to the configuration summarized in Table 3. The AdamW optimizer was employed with decoupled weight decay. Binary cross-entropy was used as the loss function. The initial learning rate was set to 2 × 10^−4^ for the CNN model and 3 × 10^−4^ for the GRU-based model. A batch size of 16 and a maximum of 20 epochs were used. Early stopping was applied based on validation the receiver operating characteristic curve (ROC-AUC) with a patience of 15 epochs and best-weight restoration. In addition, learning rate adaptation was performed using a learning rate reduction on plateau strategy (factor = 0.5, patience = 7, minimum learning rate = 10^−5^). Class imbalance was addressed using balanced class weights.

For the RF model, training was conducted using the same stratified data partitioning strategy applied to the deep learning models to ensure methodological consistency. A 15% stratified hold-out test set was reserved for final evaluation, while five-fold stratified cross-validation was performed on the remaining data to assess model stability and generalization performance. The classifier was trained directly on the normalized time–photon signal vectors without additional handcrafted feature extraction, thereby preserving fairness in the comparative framework. During cross-validation, performance metrics including validation accuracy, area under the ROC-AUC, and area under the precision–recall curve (AUPRC) were computed based on predicted class probabilities. The regularization-oriented hyper parameter configuration described in Table 2 was applied during both cross-validation and final evaluation. No additional handcrafted feature extraction was introduced, ensuring a direct comparison based solely on the original time–photon signal representation.

Overall, the unified experimental protocol ensures a consistent and methodologically controlled framework for comparative evaluation across fundamentally different learning paradigms. Model performance was assessed using accuracy, macro-averaged F1-score, ROC-AUC, and AUPRC. Cross-validation results were reported as mean performance values across folds to provide an estimate of performance stability. These complementary metrics enable both threshold-dependent (accuracy and F1-score) and threshold-independent (ROC-AUC and AUPRC) comparisons, thereby allowing a comprehensive assessment of classification performance across classical machine learning and deep learning-based approaches.

## 4. Results

This section presents the evaluation results obtained from the three learning models and summarizes their overall performance in the NLOS human detection task.

### 4.1. CNN Model Performance

Table 4 demonstrates that the CNN model exhibited highly consistent performance across the five-fold cross-validation performed on the training subset. The validation accuracy ranged from 0.956 to 0.967, and both the AUC and AUPRC values showed similar distributions, indicating a stable generalization capability across different data partitions. In addition, the validation loss showed low variability, further confirming the model stability during training.

The mean and standard deviation values across the five folds further support this finding. The validation accuracy achieved a mean of 0.960 with a standard deviation of 0.004, whereas the AUC yielded a mean of 0.972 with a standard deviation of 0.005. Similarly, the AUPRC showed a mean of 0.946, with a standard deviation of 0.015. The validation loss remained at approximately 0.183 (±0.013), indicating stable learning dynamics without meaningful fluctuations across the folds.

Table 5 summarizes the principal performance metrics obtained during the training, cross-validation, and testing phases of the CNN architecture used in this study. The close correspondence between the training and cross-validation accuracies (both 0.960), together with the high cross-validation AUC (0.972) and AUPRC (0.946) values, indicated a stable learning process and consistent discriminative capability across different data partitions. The similarity between the training and validation loss values further suggests that the model did not exhibit evident overfitting behavior during optimization.

On the independent test dataset, the model achieved an accuracy of 0.958, a ROC-AUC of 0.970, a AUPRC of 0.914, and a macro-averaged F1-score of 0.958, confirming that the learned representations generalize effectively to previously unseen samples. Overall, the results presented in Table 5 demonstrate that the CNN architecture maintains stable training dynamics and reliable classification performance across all evaluation stages.

**Figure 10:**
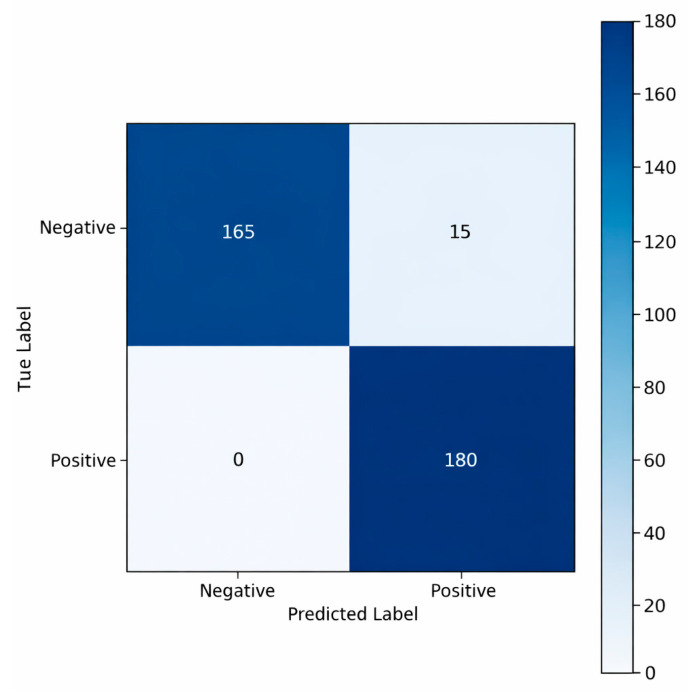
Confusion matrix of the CNN model on the test dataset.

The confusion matrix presented in Figure 10 illustrates the classification behavior of the CNN model on the independent test set consisting of 360 samples. The model correctly classified all positive-class samples (180 out of 180, indicating 100 percent sensitivity) and did not mislabel any positive instance as negative. For the negative class, 165 samples were correctly classified, whereas 15 samples were incorrectly assigned to the positive class. These results indicate that the model is highly effective in avoiding missed detections of the positive class, although it shows a limited tendency to produce false positives for the negative class. Overall, the matrix was consistent with the test accuracy and AUC scores, confirming that the CNN model demonstrated a strong discriminative performance.

### 4.2. GRU Model Performance

Table 6 presents the performance of the GRU-based RNN model during the five-fold stratified cross-validation procedure. The validation accuracy ranged from 0.869 to 0.912, whereas the AUC values varied between 0.925 and 0.947, and the AUPRC values between 0.866 and 0.921, indicating a generally stable discriminative capability across different data partitions. Although the highest performance was observed in Fold 3, the variability among the folds remained moderate, suggesting reasonable robustness to variations in the training and validation subsets.

The mean and standard deviation values further characterize the model’s overall performance. The validation accuracy was 0.886 ± 0.015, and the AUC was 0.935 ± 0.009, demonstrating a consistent learning behavior across folds. The AUPRC value of 0.896 ± 0.021 indicates that the model could capture discriminative information under class-balanced conditions. However, the comparatively higher validation loss (0.276 ± 0.029) suggests that, despite its ability to model temporal dependencies, the GRU architecture may be less effective than the CNN model in capturing the local structural patterns inherent in time–photon signal representations.

Table 7 provides a comprehensive summary of the performance metrics obtained during the training, cross-validation, and independent test stages of the GRU model. The identical training and cross-validation accuracies (both 0.886), together with the high cross-validation AUC (0.935) and AUPRC (0.896) values, indicate a stable optimization process and consistent discriminative capability across various data partitions. The close correspondence between the training and validation loss values further suggests that the model did not exhibit an evident overfitting behavior during learning.

On the independent test dataset, the GRU model achieved an accuracy of 0.886, ROC-AUC of 0.937, AUPRC of 0.920, and macro-averaged F1-score of 0.885, demonstrating that the model generalizes reasonably well to previously unseen samples. Although the GRU architecture effectively captures the temporal dependencies inherent in the NLOS time-series signals, its overall classification performance remains lower than that of the CNN model, suggesting that convolutional structures may be more effective in extracting local signal patterns in this experimental setup.

**Figure 11:**
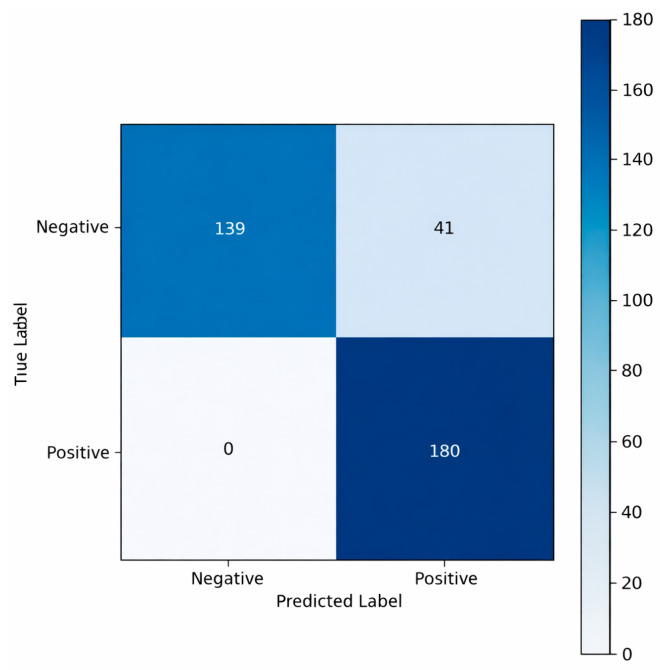
Confusion matrix of the GRU model on the test dataset.

The confusion matrix presented in Figure 11 provides a detailed illustration of the classification performance of the GRU model on the independent test dataset used. The model classified all instances of the positive class, representing the human presence condition, with perfect accuracy, producing no false negatives (FN = 0). This indicates that the GRU exhibits high sensitivity in detecting the presence of humans. However, 41 samples from the negative class were incorrectly classified as positive (FP = 41), revealing that the model made considerably more errors in distinguishing human-absent signals. This outcome suggests that although the GRU effectively captures temporal dependencies, it performs worse than the CNN in separating human-absent signals, which typically exhibit higher noise levels and greater background variability. Overall, the matrix shows that although the model is highly effective in identifying the positive class, it has limited selectivity when distinguishing the negative class.

### 4.3. Random Forest Model Performance

Table 8 shows that the RF model achieved consistently high accuracy values across both the training and validation sets during the five-fold cross-validation process. The training accuracy exceeded 0.98 in all folds, whereas the validation accuracy ranged between 0.960 and 0.978. The small gap between the training and validation performance indicates that the model does not exhibit pronounced overfitting and that its decision structure, based on statistical properties, generalizes well to the overall data distribution. However, the slight variation observed in the validation accuracy across folds suggests that the RF model may not fully capture the within-class variability arising from signal diversity.

Table 9 shows that the RF model delivers a highly accurate and stable performance when evaluated based on the cross-validation averages. The ROC-AUC value of 0.994 and the AUPRC value of 0.996, which are very close to one, indicate that the model exhibits exceptionally strong discriminative capability and maintains high sensitivity to variations arising from class distribution. The small difference between the training and validation accuracies (0.981 and 0.970) suggests that overfitting remains limited and that the model’s decision mechanism, grounded in statistical properties, generalizes well to the dataset used. These results demonstrate that the RF approach can achieve high discrimination accuracy using raw signal statistics and provides a performance that is competitive with deep learning-based methods.

Table 10 presents a consolidated performance summary of the RF model across the training, cross-validation, and independent test stages. The relatively small difference between the training accuracy (0.981) and cross-validation accuracy (0.970) suggests limited overfitting and indicates stable generalization behavior. The high cross-validation AUC (0.994) and AUPRC (0.996) values demonstrate a strong threshold-independent discriminative capability across different data partitions.

On the independent test dataset, the model achieved an accuracy of 0.972, ROC-AUC of 0.994, AUPRC of 0.995, and F1-score of 0.972, showing performance consistent with the cross-validation results. The close agreement between the validation and test metrics suggests that the model maintains a reliable classification behavior when applied to previously unseen samples. Overall, the RF model exhibited robust performance in distinguishing between classes based on the statistical characteristics of the time–photon signal representations.

**Figure 12:**
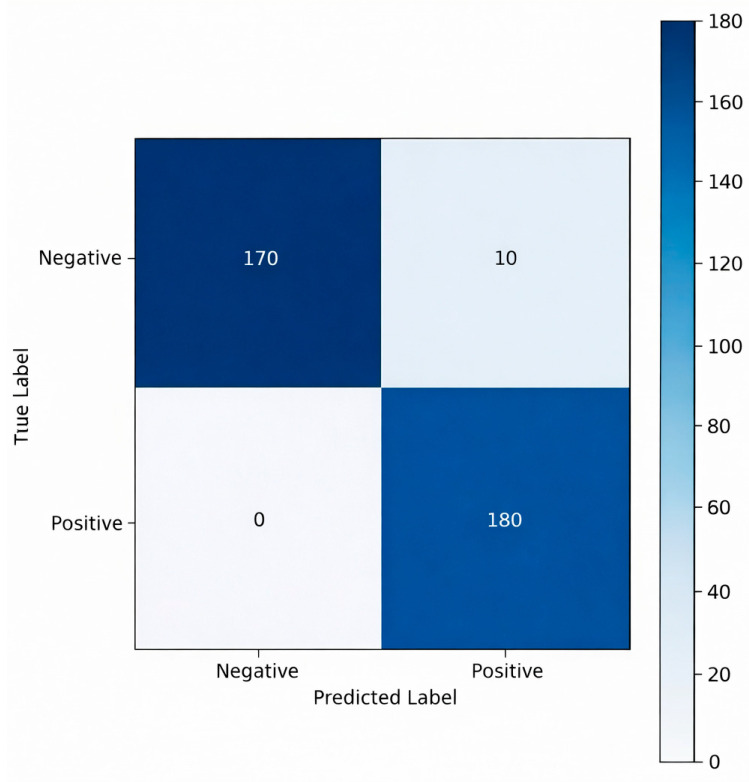
Confusion matrix of the Random Forest model on the test dataset.

The confusion matrix presented in Figure 12 provides a detailed illustration of the classification performance of the RF model on the independent test dataset. The model correctly classified all samples belonging to the human-present class without errors. For the human-absent class, only 10 samples were misclassified as positive, whereas the remaining 170 samples were correctly predicted. These results indicate that the model possesses exceptionally strong discriminative capability for the positive class while exhibiting only a minimal tendency to generate false positives in the negative class. Overall, the matrix confirms that the RF model achieves consistent class separation performance with high accuracy and low error rates.

### 4.4. Overall Comparative Performance Analysis

In this section, the validation and test metrics of the three methods evaluated in the study (CNN, GRU-based RNN, and RF) are comprehensively summarized in Table 11 and Table 12. Table 11 presents the overall performance of the models in terms of fundamental classification measures, such as accuracy, AUC, and AUPRC, whereas Table 12 provides a comparative overview of the sensitivity, specificity, and weighted F1-score values. The detailed interpretations of these tables and the underlying reasons for the performance differences among the models are discussed in Section 5.

## 5. Discussion

The comparative results presented in Table 11 and Table 12 indicate that all evaluated models achieved high performance in detecting human presence from NLOS signals. The CNN architecture effectively captured localized temporal structures and produced balanced classification results. The GRU-based RNN, despite its ability to model sequential dependencies, demonstrated comparatively lower specificity when distinguishing human-absent scenarios. Among the evaluated methods, the RF model achieved the highest validation and test performance, with ROC-AUC and AUPRC values approaching 0.99. Nevertheless, all models consistently detected human presence across the evaluated scenarios, indicating that the recorded NLOS signals contain sufficiently discriminative information for reliable classification.

The relatively strong performance of the RF model appears to be related to the structural characteristics of the preprocessed signals. After t_0_ alignment and Z-score normalization, the time–photon histograms exhibited statistically separable patterns such as peak amplitude distributions, slope variations, variance structures, and low-frequency components. In the present dataset, discriminative information is therefore primarily reflected in statistical signal properties rather than highly complex hierarchical temporal patterns. Under such conditions, ensemble tree-based models can effectively exploit these statistical feature distributions and construct robust decision boundaries.

From a signal interpretation perspective, several structural characteristics of the time–photon histograms appear to provide the most informative cues for distinguishing human-present and human-absent conditions. In particular, variations in the relative amplitude distribution of the primary and secondary reflection peaks, the temporal spread of photon arrivals, and the slope patterns surrounding dominant peaks contribute significantly to the classification decisions. In human-present scenarios, additional scattering and partial absorption introduced by the human body subtly modify the redistribution of photons within the histogram. These effects lead to consistent changes in peak intensity ratios, temporal dispersion, and local slope structures. Consequently, both the deep learning models and the RF classifier implicitly exploit these variations in photon distribution and peak morphology to differentiate between the two classes.

These observations are also consistent with the physical characteristics of the acquisition system. The integration time per scanning point was limited to 1 ms, which constrains photon count statistics and reduces the signal-to-noise ratio compared to high-time-resolution NLOS systems [8,50,57]. In addition, the effective temporal resolution of the system is influenced by detector timing jitter and laser pulse width, which limits the ability to fully resolve subtle temporal structures in the time–photon histograms. Despite these constraints, distinguishable differences between primary and secondary photon reflections were preserved, enabling reliable discrimination between human-present and human-absent conditions.

Another factor influencing model performance is the structure of the experimental dataset. The dataset used in this study consists of experimentally acquired measurements, including 1200 human-present and 300 human-absent samples. To mitigate class imbalance during training, the human-absent class was augmented using signal-preserving transformations, resulting in a balanced dataset used for model training. Each measurement required the preparation of object configurations, positioning of human subjects with different orientations and postures, and a 50 × 50 laser scanning procedure performed under dark environmental conditions. Consequently, the data acquisition process is experimentally demanding and time-consuming. Although the dataset size is moderate, the experiments were designed to incorporate multiple object scenarios, subject orientations, and postural variations in order to increase measurement diversity. Nevertheless, larger datasets may further improve model generalization capability and enable more complex learning architectures to fully exploit their representational capacity.

Taken together, these findings demonstrate that reliable human detection can be achieved using SPAD-TCSPC-based NLOS measurements under practical acquisition conditions. While the RF model exhibited slightly higher performance under the present experimental setup, this observation should not be interpreted as a general limitation of deep learning approaches. In this study, the discriminative characteristics of the time-resolved signals are relatively structured and separable, allowing tree-based models to effectively capture decision boundaries without requiring complex hierarchical feature extraction. In contrast, deep neural networks are particularly advantageous in scenarios involving higher variability, more complex temporal patterns, or less explicitly separable feature spaces. Deep neural architectures provide greater flexibility for modeling complex hierarchical dependencies and may become increasingly advantageous when larger datasets or higher temporal resolution measurements are available. Importantly, none of the evaluated models produced missed detections, which represents a critical outcome for post-disaster search and rescue applications and highlights the robustness of the proposed sensing framework.

It should be emphasized that the present study focuses on the detection of human presence under single-target NLOS conditions. The objective of this work is to investigate whether human presence can be reliably identified using SPAD-TCSPC-based measurements under controlled experimental scenarios. More complex operational tasks relevant to real search and rescue missions, such as detecting multiple individuals simultaneously, estimating the number of persons, or analyzing motion dynamics and material-related reflectivity differences (e.g., clothing properties), remain beyond the scope of the current study. Addressing these challenges would require extended measurement configurations, larger datasets, and more advanced modeling strategies, and therefore represents an important direction for future research.

In addition, the robustness of the proposed sensing framework under more complex environmental conditions should be investigated in future studies. Real-world disaster environments may contain airborne particles such as dust or smoke, as well as various sources of optical interference, which can influence photon transport and signal quality. Furthermore, heterogeneous reflective surfaces with different material properties and geometries may alter the scattering behavior of the reflected photons. Evaluating the system performance under such conditions would provide a more comprehensive understanding of the practical applicability of the proposed approach.

## 6. Conclusions and Future Work

In this study, an active NLOS sensing approach was investigated to detect the presence of a living person located outside the line of sight. An experimental system integrating a SPAD detector and a TCSPC module was developed, and a 50 × 50 laser scanning matrix was used to acquire time-resolved photon signals. Although the temporal resolution of the TCSPC system is limited by detector timing jitter and laser pulse width, the recorded signals preserved distinguishable intensity patterns associated with primary and secondary reflections. These results demonstrate that even under constrained photon statistics and acquisition conditions, time–photon histograms retain meaningful discriminative information related to human presence.

To evaluate the proposed framework, a realistic debris scenario was considered, and measurements were collected under multiple object configurations, subject postures, and orientations. Using this dataset, three different learning approaches, CNN, GRU-based RNN, and RF, were evaluated using the same preprocessing pipeline. All models successfully detected human presence in the test samples. However, differences emerged in distinguishing human-absent scenarios. The CNN achieved balanced performance with an accuracy of approximately 96%, while the GRU-based RNN reached approximately 89% accuracy. The RF model achieved the highest overall performance, exceeding 97% accuracy with the highest specificity and the lowest false-positive rate. These results indicate that the statistical variations present in NLOS time–photon signals can be effectively exploited by both deep learning and ensemble-based approaches under the current measurement conditions.

From an application perspective, the findings suggest that reliable human detection is achievable using SPAD-TCSPC-based NLOS measurements even under relatively short integration times. This capability is particularly relevant for post-disaster search-and-rescue scenarios, where rapid identification of human presence behind obstacles or debris is critical. Future research should focus on improving both the robustness and the operational scope of the proposed sensing framework. Increasing photon statistics through more sensitive SPAD detectors, longer integration times, or higher pulse energies may enhance the extraction of subtle temporal structures in the time–photon histograms. In addition, hybrid modeling strategies that combine statistical feature representations with deep learning architectures may further improve generalization performance under signal-limited conditions.

Another important direction involves evaluating the system under more complex and realistic environmental conditions. Real-world disaster environments may include airborne particles such as dust or smoke, optical interference, and heterogeneous reflective surfaces with varying material properties and geometries, all of which may influence photon propagation and signal characteristics. Investigating the impact of these factors would provide a more comprehensive assessment of the system’s practical applicability.

Finally, future studies should extend the experimental framework to more complex detection scenarios, including the simultaneous presence of multiple individuals, estimation of the number of persons, and the analysis of motion dynamics. Additional investigations should also examine the influence of clothing materials, surface reflectivity, and diverse environmental geometries on the detected photon distributions. Integrating spectral and temporal descriptors that jointly encode statistical distribution patterns and time-delay dynamics represents another promising direction for enhancing NLOS human detection systems.

## Figures and Tables

**Table 1:** Object configuration scenarios used in the experiments and their material properties relevant to reflective characteristics.

Scenario No.	Description	Expected Reflective Behavior
Scenario 0	No object	Reference measurement
Scenario 1	Wood	Moderate scattering
Scenario 2	Metal	High reflectivity
Scenario 3	Brick	Strong diffuse reflection
Scenario 4	Appliance coating	Mixed reflection
Scenario 5	PVC	Moderate reflectivity
Scenario 6	Appliance coating + PVC	Mixed reflection
Scenario 7	Wood + PVC	Combined diffuse response
Scenario 8	Brick + Wood	Strong diffuse scattering
Scenario 9	Metal + Brick	Specular + diffuse combination

**Table 2:** Random Forest hyperparameter settings and regularization rationale.

Hyperparameter	Value	Rationale/Function
Number of estimators	300	Improves ensemble stability and reduces variance.
Maximum depth	8	Limits tree complexity to reduce overfitting to noise.
Minimum samples per leaf	10	Prevents overly specific leaf rules and smooths decision boundaries.
Minimum samples per split	20	Restricts splits supported by small sample counts to reduce variance.
Maximum features	0.4	Random subspace selection (40% of features) to increase tree diversity.
Bootstrap sampling	Yes	Bagging improves robustness by training trees on resampled subsets.
Maximum samples	0.8	Each tree is trained on 80% of samples as an additional regularization.
Class weight	Balanced	Mitigates the impact of class imbalance during training.
Random state	42	Ensures reproducibility of the experiments.

**Table 3:** Optimization and training configuration of the deep learning architectures.

Parameter	Configuration	Value/Method
Optimizer	AdamW	Weight decay regularization
Initial Learning Rate	Fixed initial value	2 × 10^−4^ (CNN), 3 × 10^−4^ (GRU)
Weight Decay	Decoupled weight decay	2 × 10^−4^ (CNN), 1 × 10^−4^ (GRU)
Loss Function	Binary Cross-Entropy	Used for binary classification
Learning Rate Scheduler	Learning rate reduction on plateau	Factor = 0.5, Patience = 7, Minimum LR = 1 × 10^−5^
Early Stopping	Based on validation ROC-AUC	Patience = 15, Best weights restored
Batch Size	Mini-batch training	16
Maximum Epochs	Fixed upper limit	20
Class Balancing	Cost-sensitive learning	Balanced class weights

**Table 4:** Five-fold stratified cross-validation results of the CNN model for the binary human-present versus human-absent classification problem.

Fold	Val Accuracy	Val AUC	Val AUPRC	Val Loss
Fold 1	0.960	0.974	0.957	0.191
Fold 2	0.967	0.979	0.961	0.161
Fold 3	0.956	0.969	0.936	0.189
Fold 4	0.960	0.970	0.950	0.182
Fold 5	0.958	0.966	0.926	0.191
Mean ± Std	0.960 ± 0.004	0.972 ± 0.005	0.946 ± 0.015	0.183 ± 0.013

**Table 5:** Consolidated performance summary of the CNN model across training, cross-validation, and independent test datasets.

Metric	Value
Training Accuracy	0.960
Validation Accuracy	0.960
Validation AUC	0.972
Validation AUPRC	0.946
Training Loss	0.191
Validation Loss	0.192
Test Accuracy	0.958
Test ROC-AUC	0.970
Test AUPRC	0.914
Test F1-Score	0.958

**Table 6:** Five-fold stratified cross-validation results of the GRU-based RNN model for the binary human-present versus human-absent classification task.

Fold	Val Acc	Val AUC	Val AUPRC
Fold 1	0.869	0.929	0.899
Fold 2	0.883	0.945	0.921
Fold 3	0.912	0.947	0.911
Fold 4	0.892	0.930	0.866
Fold 5	0.875	0.925	0.885
Mean ± Std	0.886 ± 0.015	0.935 ± 0.009	0.896 ± 0.021

**Table 7:** Consolidated performance summary of the GRU-based RNN model across training, cross-validation, and independent test datasets.

Metric	Value
Training Accuracy	0.886
Validation Accuracy	0.886
Validation AUC	0.935
Validation AUPRC	0.896
Training Loss	0.278
Validation Loss	0.276
Test Accuracy	0.886
Test ROC-AUC	0.937
Test AUPRC	0.920
Test F1-Score	0.885

**Table 8:** Five-fold stratified cross-validation training and validation accuracy results of the Random Forest model for the binary human-present versus human-absent classification task.

Fold	Train Accuracy	Validation Accuracy
Fold 1	0.980	0.978
Fold 2	0.981	0.968
Fold 3	0.981	0.968
Fold 4	0.983	0.961
Fold 5	0.982	0.976
Mean ± Std	0.981 ± 0.001	0.970 ± 0.006

**Table 9:** Five-fold cross-validation mean performance metrics of the Random Forest model.

Metric	Mean
Train Accuracy	0.981
Validation Accuracy	0.970
Validation ROC-AUC	0.994
Validation AUPRC	0.996

**Table 10:** Consolidated performance summary of the Random Forest model across training, cross-validation, and independent test datasets.

Metric	Value
Training Accuracy (Mean Train Acc)	0.981
Validation Accuracy (Mean Val Acc)	0.970
Validation AUC (Mean Val AUC)	0.994
Validation AUPRC (Mean Val AUPRC)	0.996
Test Accuracy	0.972
Test ROC-AUC	0.994
Test AUPRC	0.995
Test F1-Score	0.972

**Table 11:** Comparative summary of the validation and test performance of the models.

Model	Val Acc (Mean)	Val AUC (Mean)	Val AUPRC (Mean)	Test Acc	Test F1 (Weighted)	Test ROC-AUC	Test AUPRC
CNN	0.960	0.972	0.946	0.958	0.958	0.970	0.914
GRU	0.886	0.935	0.896	0.886	0.885	0.937	0.920
RF	0.970	0.994	0.996	0.972	0.972	0.994	0.995

**Table 12:** Comparison of the sensitivity, specificity, and weighted F1-score performance of the models.

Model	Sensitivity (Human-Present)	Specificity (Human-Absent)	F1 (Weighted)
CNN	1.000	0.917	0.958
GRU	1.000	0.772	0.885
RF	1.000	0.944	0.972

## Data Availability

Data are available from the corresponding author upon reasonable request. The dataset contains controlled laboratory measurements and cannot be publicly released at this time.

## References

[1] Zheng Y., Wang W., Zhang C., Zhang Y., Zhang Q., Li L. (2024). Converting non-confocal measurements into semi-confocal ones with timing-accuracy improving for non-line-of-sight imaging. Opt. Lasers Eng..

[2] Lei X., He L., Tan Y., Wang K.X., Wang X., Du Y., Fan S., Yu Z. Direct object recognition without line-of-sight using optical coherence. Proceedings of the IEEE/CVF Conference on Computer Vision and Pattern Recognition (CVPR).

[3] He J., Wu S., Wei R., Zhang Y. (2022). Non-line-of-sight imaging and tracking of moving objects based on deep learning. Opt. Express.

[4] Wang C., Wang X., Fang Y., Yan C., Zhang X., Zuo Y. (2024). Event-enhanced passive non-line-of-sight imaging for moving objects with physical embedding. IEEE Sens. J..

[5] Riccardo S., Conca E., Sesta V., Tosi A. Fast-gated 16 × 16 SPAD array with on-chip 6 ps TDCs for non-line-of-sight imaging. Proceedings of the 2021 IEEE Photonics Conference (IPC).

[6] Zheng S., Liao M., Wang F., He W., Peng X., Situ G. (2021). Non-line-of-sight imaging under white-light illumination: A two-step deep learning approach. Opt. Express.

[7] Lindell D.B., Wetzstein G., Koltun V. Acoustic non-line-of-sight imaging. Proceedings of the IEEE/CVF Conference on Computer Vision and Pattern Recognition (CVPR).

[8] Isogawa M., Yuan Y., O’Toole M., Kitani K.M. Optical Non-Line-of-Sight Physics-Based 3D Human Pose Estimation. Proceedings of the IEEE/CVF Conference on Computer Vision and Pattern Recognition (CVPR).

[9] Feng X., Gao L. (2021). Toward non-line-of-sight videography. Opt. Photonics News.

[10] King E.A., Tatoglu A., Iglesias D., Matriss A. (2021). Audio-visual based non-line-of-sight sound source localization: A feasibility study. Appl. Acoust..

[11] An I., Son M., Manocha D., Yoon S.-E. Reflection-aware sound source localization. Proceedings of the 2018 IEEE International Conference on Robotics and Automation (ICRA).

[12] Çalışan M., Olgun N., Gürgöze G., Doğan F., Nergiz M., Türkoğlu İ. Eşzamanlı ses sinyalleri kullanılarak görüş hattı dışı ortamlarda nesne sınıflandırması. Proceedings of the 13th International Conference on Scientific Research.

[13] Schulz Y., Mattar A.K., Hehn T.M., Kooij J.F.P. (2021). Hearing what you cannot see: Acoustic vehicle detection around corners. IEEE Robot. Autom. Lett..

[14] Bouman K.L., Ye V., Yedidia A.B., Durand F., Wornell G.W., Torralba A., Freeman W.T. Turning Corners into Cameras: Principles and Methods. Proceedings of the 2017 IEEE International Conference on Computer Vision (ICCV).

[15] Seidel S.W., Murray-Bruce J., Ma Y., Yu C., Freeman W.T., Goyal V.K. (2021). Two-Dimensional Non-Line-of-Sight Scene Estimation From a Single Edge Occluder. IEEE Trans. Comput. Imaging.

[16] AYedidia B., Baradad M., Thrampoulidis C., Freeman W.T., Wornell G.W. Using Unknown Occluders to Recover Hidden Scenes. Proceedings of the IEEE/CVF Conference on Computer Vision and Pattern Recognition.

[17] Torralba A., Freeman W.T. Accidental pinhole and pinspeck cameras: Revealing the scene outside the picture. Proceedings of the 2012 IEEE Conference on Computer Vision and Pattern Recognition (CVPR).

[18] Boger-Lombard J., Katz O. (2019). Passive optical time-of-flight for non-line-of-sight localization. Nat. Commun..

[19] Beckus A., Tamasan A., Atia G.K. (2019). Multi-modal non-line-of-sight passive imaging. IEEE Trans. Image Process..

[20] Luesia-Lahoz P., Cartiel S., Muñoz A. (2026). Stereo Non-Line-of-Sight Imaging. Vis. Comput..

[21] Henley C., Maeda T., Swedish T., Raskar R. (2020). Imaging Behind Occluders Using Two-Bounce Light. Proceedings of the Computer Vision—ECCV 2020, Lecture Notes in Computer Science.

[22] Liu P., Yu Y., Pan Z., Peng X., Li R., Wang Y., Yu J., Li S. (2023). HiddenPose: Non-Line-of-Sight 3D Human Pose Estimation. IEEE Trans. Pattern Anal. Mach. Intell..

[23] Geng R., Hu Y., Chen Y. (2023). Recent advances on non-line-of-sight imaging: Conventional physical models, deep learning, and new scenes. IEEE Trans. Comput. Imaging.

[24] Velten A., Willwacher T., Gupta O., Veeraraghavan A., Bawendi M.G., Raskar R. (2012). Recovering three-dimensional shape around a corner using ultrafast time-of-flight imaging. Nat. Commun..

[25] Arellano V., Gutierrez D., Jarabo A. (2017). Fast back-projection for non-line-of-sight reconstruction. Opt. Express.

[26] Heide F., Xiao L., Heidrich W., Hullin M.B. Diffuse mirrors: 3D reconstruction from diffuse indirect illumination using inexpensive time-of-flight sensors. Proceedings of the 2014 IEEE Conference on Computer Vision and Pattern Recognition (CVPR).

[27] Liu X., Bauer S., Velten A. (2020). Phasor field diffraction-based reconstruction for fast non-line-of-sight imaging systems. Nat. Commun..

[28] Lindell D.B., Wetzstein G., O’Toole M. (2019). Wave-based non-line-of-sight imaging using fast f-k migration. ACM Trans. Graph..

[29] O’Toole M., Lindell D.B., Wetzstein G. (2018). Confocal non-line-of-sight imaging based on the light-cone transform. Nature.

[30] Ye J.-T., Huang X., Li Z.-P., Xu F. (2021). Compressed sensing for active non-line-of-sight imaging. Opt. Express.

[31] Liu X., Velten A. The role of Wigner distribution function in non-line-of-sight imaging. Proceedings of the 2020 IEEE International Conference on Computational Photography (ICCP).

[32] Isogawa M., Chan D., Yuan Y., Kitani K., O’Toole M., Vedaldi A., Bischof H., Brox T., Frahm J.M. (2020). Efficient Non-Line-of-Sight Imaging from Transient Sinograms. Computer Vision—ECCV 2020. ECCV 2020. Lecture Notes in Computer Science.

[33] Gariepy G., Krstajić N., Henderson R., Li C., Thomson R.R., Buller G.S., Heshmat B., Raskar R., Leach J., Faccio D. (2015). Single-photon sensitive light-in-fight imaging. Nat. Commun..

[34] Nam J.H., Brandt E., Bauer S., Liu X., Renna M., Tosi A., Sifakis E., Velten A. (2021). Low-latency time-of-flight non-line-of-sight imaging at 5 frames per second. Nat. Commun..

[35] Renna M., Nam J.H., Buttafava M., Villa F., Velten A., Tosi A. (2020). Fast-gated 16 × 1 SPAD array for non-line-of-sight imaging applications. Instruments.

[36] Della Rocca F.M., Mai H., Hutchings S.W., AI Abbas T., Buckbee K., Tsiamis A., Lomax P., Gyongy I., Dutton N.A., Henderson R.K. (2020). A 128 × 128 SPAD Motion-Triggered Time-of-Flight Image Sensor with in-Pixel Histogram and Column-Parallel Vision Processor. IEEE J. Solid-State Circuits.

[37] Wu C., Liu J., Huang X., Li Z.-P., Yu C., Ye J.-T., Zhang J., Zhang Q., Dou X., Goyal V.K. (2021). Non–line-of-sight imaging over 1.43 km. Proc. Natl. Acad. Sci. USA.

[38] Princeton Computational Imaging Lab Low-Cost SPAD Sensing for Non-Line-of-Sight Tracking, Material Classification and Depth Imaging. https://light.princeton.edu/publication/cheapspad/.

[39] Ballester M., Wang H., Li J., Cossairt O., Willomitzer F. (2024). Single-shot synthetic wavelength imaging: Sub-mm precision ToF sensing with conventional CMOS sensors. Opt. Lasers Eng..

[40] Olgun N., Çalışan M., Türkoğlu İ. Non-line-of-sight human detection using laser signals. Proceedings of the 4th International Conference on Advanced Technologies, Computer Engineering and Science (ICATCES’25).

[41] Liao Z., Jiang D., Liu X., Velten A., Ha Y., Lou X. (2022). FPGA accelerator for real-time non-line-of-sight imaging. IEEE Trans. Circuits Syst. I Regul. Pap..

[42] Maeda T., Satat G., Swedish T., Sinha L., Raskar R. (2019). Recent advances in imaging around corners. Imaging Systems for Deep Learning.

[43] Chen Z., Chen Z., Li Z., Tang W., Zhang D., Wang T., Liu Q., Yu T. (2026). Canny operator-based artifact identification and suppression for non-line-of-sight imaging. Opt. Laser Technol..

[44] Liu X., Guillén I., La Manna M., Nam J.H., Reza S.A., Le T.H., Gutierrez D., Jarabo A., Velten A. (2019). Non-line-of-sight imaging using phasor-field virtual wave optics. Nature.

[45] Tsai C.-Y., Sankaranarayanan A.C., Gkioulekas I. Beyond volumetric albedo-A surface optimization framework for non-line-of-sight imaging. Proceedings of the IEEE Conference on Computer Vision and Pattern Recognition (CVPR).

[46] Reza S.A., La Manna M., Bauer S., Velten A. (2019). Phasor field waves: A Huygens-like light transport model for non-line-of-sight imaging applications. Opt. Express.

[47] Cao Y., Liang R., Zhu W., Zhao B., Chen H., Shen L., Yang J., Cao Y., Chen J., Li X. (2023). Dynamic-excitation-based steady-state non-line-of-sight imaging via multi-branch convolutional neural network. Opt. Lasers Eng..

[48] Caramazza P., Boccolini A., Buschek D., Hullin M., Higham C.F., Henderson R., Murray-Smith R., Faccio D. (2018). Neural network identification of people hidden from view with a single-pixel, single-photon detector. Nat. Photonics.

[49] Hsu Y.-L., Yang C.-L., Chang C.-W., Lai H.-C. (2019). A novel CNN-based framework for classification of signal quality and sleep position from a capacitive ECG measurement. Sensors.

[50] Li C., Chen J., Yang C., Yang J., Liu Z., Davari P. (2023). Convolutional neural network-based transformer fault diagnosis using vibration signals. Sensors.

[51] Larsen M.L.V., Nielsen J.P., Pedersen L.J. (2018). A combined deep learning GRU-autoencoder for the early detection of respiratory disease in pigs. Sensors.

[52] Metzler C.A., Heide F., Rangarajan P., Balaji M.M., Viswanath A., Veeraraghavan A., Baraniuk R.G. (2020). Deep-inverse correlography: Towards real-time high-resolution non-line-of-sight imaging. Optica.

[53] Lin Y., Mos P., Ardelean A., Bruschini C., Charbon E. (2024). Coupling a recurrent neural network to SPAD TCSPC systems for real-time fluorescence lifetime imaging. Sci. Rep..

[54] Li Y., Peng J., Ye J., Zhang Y., Xu F., Xiong Z. NLOST: Non-line-of-sight imaging with Transformer. Proceedings of the IEEE/CVF Conference on Computer Vision and Pattern Recognition (CVPR).

[55] Shen S., Wang Z., Peng X., Xia S., Li R., Li S., Yu J. (2025). MARMOT: Masked autoencoder for modeling transient imaging. arXiv.

[56] Faccio D., Velten A., Wetzstein G. (2020). Non-line-of-sight imaging. Nat. Rev. Phys..

[57] Chandran S., Jayasuriya S. (2019). Adaptive lighting for data-driven non-line-of-sight 3D localization and object identification. arXiv.

